# Clinical guided computer tomography decisions are advocated in potentially severely injured trauma patients: a one-year audit in a level 1 trauma Centre with long pre-hospital times

**DOI:** 10.1186/s13049-019-0692-5

**Published:** 2020-01-10

**Authors:** Anna Bågenholm, Trond Dehli, Stig Eggen Hermansen, Kristian Bartnes, Marthe Larsen, Tor Ingebrigtsen

**Affiliations:** 1 Department of Clinical Medicine, Faculty of Health Science, UiT-The Artic University of Norway, PO box 6050 Langnes, N-9037 Tromsø, Norway; 2 Department of Radiology, University Hospital of North Norway, Sykehusveien 38, PO box 103, N-9038 Tromsø, Norway; 3 Department of Gastrointestinal Surgery, University Hospital of North Norway, PO box 103, N-9038 Tromsø, Norway; 4 Department of Cardiothoracic and Vascular Surgery, University Hospital of North Norway, PO box 103, N-9038 Tromsø, Norway; 5 Centre for Quality Improvements and Development, University Hospital of North Norway, PO box 103, N-9038 Tromsø, Norway; 6 Department of Neurosurgery, ENT and Ophthalmology, University Hospital of North Norway, PO box 103, N-9038 Tromsø, Norway

**Keywords:** Trauma audit, Trauma population, Injury, Multi trauma, Diagnostic imaging, Whole body computer tomography, Decision tool, Dose limitation

## Abstract

**Background:**

The International Commission on Radiological Protection’s (ICRP) justification principles state that an examination is justified if the potential benefit outweighs the risk for radiation harm. Computer tomography (CT) contributes 50% of the radiation dose from medical imaging, and in trauma patients, the use of standardized whole body CT (SWBCT) increases. Guidelines are lacking, and reviews conclude conflictingly regarding the benefit. We aimed to study the degree of adherence to ICRP’s level three justification, the individual dose limitation principle, in our institution.

**Methods:**

This is a retrospective clinical audit. We included all 144 patients admitted with trauma team activation to our regional Level 1 trauma centre in 2015. Injuries were categorized according to the Abbreviated Injury Scale (AIS) codes. Time variables, vital parameters and interventions were registered. We categorized patients into trauma admission SWBCT, selective CT or no CT examination strategy groups. We used descriptive statistics and regression analysis of predictors for CT examination strategy.

**Results:**

The 144 patients (114 (79.2%) males) had a median age of 31 (range 0–91) years. 105 (72.9%) had at least one AIS ≥ 2 injury, 26 (18.1%) in more than two body regions. During trauma admission, at least one vital parameter was abnormal in 46 (32.4%) patients, and 73 (50.7%) underwent SWBCT, 43 (29.9%) selective CT and 28 (19.4%) no CT examination. No or only minor injuries were identified in 17 (23.3%) in the SWBCT group. Two (4.6%) in the selective group were examined with a complement CT, with no new injuries identified. A significantly (*p < 0.001*) lower proportion of children (61.5%) than adults (89.8%) underwent CT examination despite similar injury grades and use of interventions. In adjusted regression analysis, patients with a high-energy trauma mechanism had significantly (*p = 0.028*) increased odds (odds ratio = 4.390, 95% confidence interval 1.174–16.413) for undergoing a SWBCT.

**Conclusion:**

The high proportion of patients with no or only minor injuries detected in the SWBCT group and the significantly lower use of CT among children, indicate that use of a selective CT examination strategy in a higher proportion of our patients would have approximated the ICRP’s justification level three, the individual dose limitation principle, better.

## Background

Medical imaging adds 1.1 millisievert (mSv) to Europeans and 3 mSv to Americans to the average natural background dose of 2–3 mSv per year. Approximately 50% of this dose comes from computer tomography (CT) [1, 2]. The International Commission on Radiological Protection (ICRP) introduced a system for dose limitation to humans in 1977. The system has three justification levels [3, 4]. Level one deals with the use of radiation in medicine in general, level two with specified procedures, and level three with the application of a procedure to an individual. At level three, an examination is justified if the given dose gains the patient more than the potential ionizing harm [3–5]. It is established that young and healthy persons have increased risk for long-time harm after ionizing radiation [3, 4, 6, 7].

Recently, dose limitation to individuals has been emphasized due to the increased use of CT, for example in the Triple “A” (awareness, appropriateness, and audit) advice from 2009 [8], the Nordic radiation protecting agencies’ statement concerning high CT examination use from 2012 [9] and the European society of radiology’s clinical decision support for imaging referral “iGuide” from 2016 [10].

A trauma system should therefore diagnose the mostly young and healthy trauma patients with an individually optimized diagnostic strategy [3–5, 7–11]. During the last 20 years, the use of standardized whole body CT (SWBCT) has increased due to better availability and functionality of CT machines and numerous publications recommending this examination as routine [12–18]. Generally accepted guidelines for use of SWBCT in trauma patients are, however, lacking [19–23]. Reviews assessing survival after treatment for trauma conclude conflictingly regarding the benefit of SWBCT [24–27]. The only prospective randomised study of immediate SWBCT compared to individual imaging after a clinical examination was published in 2016 by Sierink et al. [28]. They found no difference in mortality, but showed increased radiation exposure in the immediate SWBCT group. Few studies have specifically assessed whether the use of SWBCT fulfils the individual dose limitation criterion for justification at ICRP’s level three [29].

SWBCT examination in potentially severely injured patients gives the trauma team a tool for fast decision making on intervention, identifies injuries not suspected and facilitates patient logistics [14, 30–34]. Accordingly, in our institution, the use of SWBCT has increased over the last fifteen years. It is unclear, however, whether the present use is in accordance with ICRP’s level three justification, the individual dose limitation principle.

## Methods

### Study type and aim of the study

This is a retrospective clinical audit [35, 36]. We aimed to study the degree of adherence to ICRP’s level three justification, the individual dose limitation principle, in our institution. To achieve this, we describe the identified injuries, and the use of CT examinations and interventions in suspected severely injured trauma patients. In addition, we analyse associations between parameters that could influence CT use during trauma admissions, and the observed actual use of CT.

### Study region

The northern Norway health region has 486,792 inhabitants (2015) spread out over a rural area of 257,450 km^2^ (1.9 inhabitants/km^2^) [37, 38]. The University Hospital of North Norway, Tromsø campus (UNN) is defined as the regional Level 1 trauma centre, by the Norwegian Trauma system. UNN has approximately 150 trauma team activations (TTA) per year, and supports ten referring hospitals, of which none has CT in the emergency department (ED). The region is served by an advanced fixed and rotor wing air ambulance service [39]. The smallest and most remote referring hospital, located on the Spitsbergen islands, has no CT and is situated 2.5 h away with fixed wing propel air-plane.

The region has predefined criteria for TTA [40] and follows the Advanced Trauma Life Support® system [41]. Decision on the use of diagnostic CT is on the discretion of the trauma surgeon in charge. The technical protocol for SWBCT in adults (> 16 years) is standardized. Patients may undergo CT and interventions during three phases: trauma admission one at a referring hospital or at UNN for patients transported directly to the Level 1 trauma centre (phase one), trauma admission two for referred patients (phase two) or during the subsequent hospital stay (phase three). The total hospitalization includes all three phases.

### Inclusion criteria and data collection

Trauma registrars continuously survey emergency admissions and prospectively register all trauma patients fulfilling predefined criteria in the national trauma registry [42]. In the present study, we included all patients registered with a TTA at UNN from 01.01 to 31.12.2015. There were no exclusion criteria.

The first author thereafter manually retrieved and registered all study data from pre- and intra-hospital electronic health records, including the radiology information system (RIS) and the radiology examinations and logs in the picture archiving and communication system (PACS). Study data entry continued until death, or discharge home or to rehabilitation.

### Injury code identification and estimation of injury severity

Injuries were categorized with Abbreviated Injury Scale (AIS) codes [43]. We used the AIS 2005 update 2008 manual [44]. The first author and another AIS-certified physician, employed as trauma registry coder at UNN, made a consensus coding on all injury codes. The AIS codes are presented as total number of codes in the population. One code is a combination of a six-digit pre-dot anatomical code and a one digit post dot severity score ranked from one (minor) to six (lethal). We report the total number of codes with severity scores ≥2 for the population.

Total injury severity per patient is estimated with the Injury Severity Score (ISS) [45] and the New ISS (NISS) [46]. ISS uses six body regions. Patients with ISS or NISS > 15 were defined as severely and those with scores between 4 and 15 as moderately injured. To differentiate patients with no injuries or injuries not detectable with CT (AIS 1) from those detectable (AIS ≥ 2), we dichotomized patients as ISS 0–3 or 4–75. Total severity estimates per patient were also reported as the number of ISS regions per patient with identified AIS ≥ 2 codes. We defined polytrauma as AIS > 2 in at least two of the six ISS body regions [47, 48].

### Time variables

We registered the time span from the accident to arrival in hospital, and to the start of the first CT scan.

### Vital parameters (adults)

We registered the vital parameters (systolic blood pressure (SBP) (mmHg), heart rate (HR) (beats/minute), respiratory rate (RR) (breath/minute) and Glasgow coma scale (GCS) score) as continuous variables at the accident site, during trauma admissions immediately before the first possible CT examination strategy decision, and calculated shock index (SI) = HR/SBP [49–51].

SBP, RR and GCS were dichotomised as normal or abnormal according to the Revised Trauma Score standards [52], and HR according to our trauma team activation manual [40]. SI was categorized as abnormal if > 0.9 [50, 51].

We merged the dichotomized vital parameters SBP, HR, RR and GCS score into a new parameter (merged vital parameter). We defined it as abnormal if one or more of the four were abnormal, also if only one of the four was documented. If one was normal and three were undocumented or if all were undocumented, the merged vital parameter was registered as missing. All other combinations were defined as normal.

Arterial haemoglobin, lactate and base excess, obtained immediately before the first possible CT examination strategy decision, were recorded as continuous parameters. Lactate and base excess were also dichotomized as normal or abnormal according to the reference in our institution [53, 54].

### Vital parameters (children)

We registered the same vital parameters for children. GCS is validated for use in children with the same standards as adults and used in this study [55]. We dichotomised SBP, HR and RR as normal or abnormal according to the Norwegian modified paediatric early warning score [56–59], and defined SI as abnormal if > 1.22 up to six years, > 1.0 between six and twelve years and > 0.9 above twelve years [60, 61].

### CT examinations

We categorised the patients into three trauma admission CT examination strategy groups; SWBCT, selective CT or no CT. The SWBCT protocol includes scans of the caput and neck without intravenous contrast, scan of the chest with intravenous contrast in the arterial phase (including the spleen), and scan of the abdomen/pelvis with intravenous contrast in the portal venous phase. We defined a selective CT as an examination excluding one or more of the four SWBCT body part scans. It can include extremity scans. In the no CT group, the cause for not undergoing CT examination was categorised as either “no indication for CT” or “patient too hemodynamically unstable for CT”.

CT examinations were also categorised as ordered during the trauma admissions or during the subsequent hospital stay. If the trauma admission took place in the operating room (OR), as for some of the hypothermia and burn patients, CT examinations ordered afterwards were registered as done during trauma admission, according to local guidelines. When patients were transported directly to the OR due to hemodynamic instability of other reasons, CT examinations ordered afterwards were registered as done during the subsequent hospital stay.

We defined a duplicated CT examination as a body part examined more than once, and a complement CT as a body part scan done during the subsequent hospital stay for a body part not examined during the trauma admissions.

We categorised patients into three groups based on the trauma admission CT examination findings. High injury grade group was defined as AIS ≥ 2 injuries identified in two or more SWBCT body part scans, moderate injury grade group as AIS ≥ 2 injuries in one body part and low injury grade group as either AIS 1 injuries or no injuries.

### Interventions

We defined interventions as action taken to improve the outcome of an injury, or to prevent it from getting worse. For each patient, we registered whether the patient had undergone intervention(s) or not, and eventually the types and number of interventions. Interventions were categorised as active procedures or conservative treatment decisions, such as for example observations. Repeated interventions for the same injury were registered as one.

Emergency interventions were defined as those listed in the Norwegian trauma registry manual [42] and done within 24 h after the accident. In addition, we defined active internal and external rewarming as emergency interventions. Intubation is not listed in the manual, and was therefore not registered as an emergency intervention, but we registered whether patients were intubated pre-hospitally or within the first 24 h after admission. We also registered the total number of interventions per patient done during hospitalization in areas examined with a trauma admission CT.

### Statistics

Continuous variables are presented with mean and standard deviation (SD) or median and lower (Q1) and upper quartile (Q3) depending on the distribution of the variable. Categorical variables are presented with frequencies and percentages. Group differences are tested with independent-t-test for continuous variables and chi-square test or Fisher’s exact test for categorical variables. Associations between clinical parameters assessable for the 113 adult patients examined in the ED before trauma admission CT strategy decision, and the use of SWBCT versus a selective or no CT approach were analysed with logistic univariable and multivariable regression. Unadjusted and adjusted odds ratios are presented with 95% confidence interval (CI) and *p*-values. Five hemodynamically unstable adults sent directly to OR and the children were excluded from this analysis. *P*-values < 0.05 were considered statistically significant. IBM SPSS 24 was used to analyse the data.

## Results

### Demographics

Table 1 displays characteristics for the 144 patients.

**Table 1:** Characteristics of the trauma population (*n* = 144)

Characteristics	
Male sex, n (%)	114 (79.2)
Tourist, n (%)	28 (19.4)
Age, years in median (Q1, Q3)	31 (19, 49)
Age groups, n (%)	
< 5	9 (6.3)
5–16	17 (11.8)
> 16	118 (81.9)
Transport to first hospital by	
Ambulance helicopter, n (%)	80 (55.6)
Fixed wing air ambulance, n (%)	9 (6.2)
Road ambulance, n (%)	53 (36.8)
Private transportation, n (%)	2 (1.4)
Trauma mechanism	
Penetrating traumas, n (%)	5 (3.5)
Blunt, n (%)	139 (96.5)
Road traffic, n (%)	63 (45.3)
Snowmobile, n (%)	11 (7.9)
Falls, n (%)	31 (22.3)
Hit by blunt object, n (%)	13 (9.4)
Explosion/fire, n (%)	8 (5.7)
Avalanches and/or hypothermia, n (%)	8 (5.8)
Other causes, n (%)	5 (3.6)
Transferred from other hospitals, n (%)	36 (25.0)
ISS, (Q1, Q3, Range)	9 (2, 22, 0–59)
NISS, (Q1, Q3, Range)	12 (3, 27, 0–66)
ISS > 15, n (%)	52 (36.1)
ISS > 15 among 26 children ≤16 years, n (%)	5 (19.2)
ISS > 15 among 118 adults, n (%)	47 (39.8)
NISS > 15, n (%)	64 (44.4)
Length of stay, median days (Q1, Q3)	4 (1.2, 11.5)
Total hospitalization > 20 days, n (%)	20 (13.9)
30-day mortality, n (%)	10 (6.9)

*Q1* lower quartile*, Q3 upper* quartile, *ISS* Injury Severity Score, *NISS* New Injury Severity score

ISS and NISS were positively skewed. Twenty-four (16.7%) had polytrauma. The 10 patients dying within 30 days had ISS between 22 and 45, and seven were polytraumatized. Three patients (2.1%) died within 24 h after the accident.

### Identified injuries

We identified 766 AIS injury codes in 138 (95.8%) of the 144 patients. The majority 469 (61.2%) were AIS ≥2 injuries in 105 (72.9%) patients, of which 54 (37.5%) had at least one AIS ≥ 2 injury in one, 25 (17.4%) in two, and 26 (18.1%) in three or more ISS body regions.

### Time variables

Median time from the accident to arrival in the first hospital with CT possibility was 1 h and 54 min (Q1 = 1.0, Q3 = 2.7 h). Fifty-two (36.1%) patients arrived within 1 h and 30 min and 21 (14.6%) after five or more hours. Median time from the accident to start of the first CT examination (*n* = 116 patients) was 2 h and 36 min (Q1 = 1.8, Q3 = 4.0 h), and median time from arrival in hospital to start of the first CT 43 min (Q1 = 0.6, Q3 = 0.9 h). Twenty (17.2%) had the examination within 1 h and 30 min after the accident and 23 (19.8%) after five or more hours.

### Vital parameters

Table 2 displays the parameters registered at the accident site and immediately before the first possibility to order a CT examination, and the proportions with abnormal findings. These patient specific data were available to the trauma surgeon before a decision about trauma admission CT examination strategy was reached.

**Table 2:** First documented vital parameters at accident site and during first trauma admission with CT possibility

First documented vital parameters in patient record or assessed/calculated value from the documented parameters	Accident site	TA with first CT possibility
	n = 144	n = 144
Heart rate, n (%)	112 (77.8)	140 (97.2)
Abnormal (< 40 or > 130 beats/minute ^a^), n (%)	7 (6.2)	5 (3.6)
Systolic blood pressure, n (%)	107 (74.3)	139 (96.5)
Abnormal (< 90 mmHg ^a^), n (%)	10 (9.3)	6 (4.3)
Respiratory rate_,_ n (%)	87 (60.4)	115 (79.9)
Abnormal (< 10 or > 29 breaths/minute ^a^), n (%)	20 (23.0)	12 (10.4)
Glasgow Coma Scale value, (%)	126 (87.5)	133 (92.4)
Admitted intubated in trauma admission just before CT decision		11 (7.6)
Abnormal (< 13 at accident site, < 13 or intubated in TA), n (%)	41 (28.5)	35 (23.4)
Shock index, heart rate/systolic blood pressure, n (%)	99 (68.8)	138 (95.8)
Abnorma, (> 0.9, > 1 6–12 years, > 1.22 < 6 years) n (%)	10 (6.9)	18 (12.5)
Blood lactate, n (%)		90 (62.5)
Abnormal (> 1.8 mmol/l), n (%)		35 (24.3)
Abnormal and > 32 degree Celsius, n (%)		30 (20.8)
Blood base excess, n (%)		91 (63.2)
Abnormal (< −3.3 to > 3.3 mmol/l), n (%)		29 (20.1)

*CT* Computer tomography, *TA* trauma admission, ^a^ abnormal children values dichotomized according to the Norwegian modified paediatric early warning scores normal values

The merged vital parameter was abnormal in 46 (32.4%) of 142 patients immediately before trauma admission CT examination strategy decision. The proportion with abnormal SI (21.7% versus 8.3%, *p = 0.017*)*,* base excess (41.3% versus 7.0%, *p < 0.001*), lactate (41.3% versus 16.7%, *p =* 0.06) was higher among these 46 patients compared to those with a normal merged vital parameter.

### CT examinations

Among the 144 patients, 116 (80.5%) underwent CT examination during the trauma admissions. Seventy-three (62.9%) underwent a SWBCT and 43 (37.1%) a selective CT examination among the 116 with CT. During the total hospitalization, 122 (84.7%) underwent CT. Table 3 shows the distribution of patients in the three trauma admission CT examination strategy groups.

**Table 3:** CT examinations in patients admitted with TTA

	< 5 years n, (%)	5–16 years n, (%)	> 16 years n, (%)	Total, n (%)
Trauma admission CT examination strategy groups				
Standardized whole body CT	1 (11.1)	6 (35.3)	66 (55.9)	73 (50.7)
Duplicated CT ^a^ during subsequent hospital stay	0 (0.0)	2 (33.3)	34 (51.5)	36 (49.3)
Selective CT	0 (0.0)	7 (41.2)	36 (30.5)	43 (29.9)
Duplicated CT ^a^ during subsequent hospital stay	0 (0.0)	1 (14.3)	8 (22.2)	9 (20.9)
Complement CT ^b^ during subsequent hospital stay	0 (0.0)	0 (0.0)	2 (5.6)	2 (4.6)
No CT	8 (88.9)	4 (23.5)	16 (13.6)	28 (19.4)
Complement CT ^b^ during subsequent hospital stay	1 (12.5)	1 (25.0)	4 (25.0)	6 (21.4)
Total, n (%)	9 (100)	17 (100)	118 (100)	144 (100)

*CT* Computer Tomography, *TTA* trauma team activation, ^a^ CT of a body part examined during trauma admission and at least once during the subsequent hospital stay, ^b^ CT of a body part not examined during trauma admission but during the subsequent hospital stay

In the selective CT group, two adults underwent a complement CT without detection of previously undiagnosed injuries.

Among the 28 patients in the no CT group, five (17.8%) went directly to the OR due to hemodynamic instability; four of these were adults examined with a complement CT during the subsequent hospital stay. The remaining 23 (82.1%) had symptoms and/or injuries for which the surgeon decided that a CT was unnecessary or that it was safe to spare the patient for the ionizing radiation. Five had an ISS > 15, seven between 4 and 15 and eleven < 4. Two children underwent a complement CT without detection of previously undiagnosed injuries.

The proportion with abnormal findings on the merged vital parameter immediately before the CT examination strategy decision was significantly lower in the selective CT group (14.3%) compared to the SWBCT group (39.7%, *p = 0.004)* and the no CT group (40.7%, *p = 0.013*). In the no CT group, seven (31.8%) of the 23 patients for whom CT was considered unnecessary and four (80.0%) of the five who went directly to OR had an abnormal merged vital parameter.

Table 4 displays the use of duplicated CT during the sub-sequent hospital stay. None of the control CT examinations led to active interventions.

**Table 4:** Duplicated CT examination per patient during the subsequent hospital stay after a TA CT

CT type during trauma admission	SWBCT *n* = 36	Selective CT *n* = 9
Control CT for findings seen on trauma admission CT ^a^, n (%)	28 (77.8)	7 (77.8)
New finding identified ^b^, not described on TA CT	4 (14.3)	1 (16.7)
Active intervention chosen	0 (0)	0 (0.0)
Conservative intervention chosen	4 (100.0)	1 (100.0)
New CT because of new vital indication	8 (22.2)	2 (22.2)
New finding identified ^b^, not described on TA CT	4 (50.0)	2 (50.0)
Active intervention	3 (75..0)	2 (100.0)
Conservative intervention	1 (25.0)	0 (0.0)

*CT* Computer Tomography, *TA* trauma admission*, SWBCT* Standardized whole body CT, ^a^ The main reason per person registered, ^b^ The main finding registered

### Interventions

Table 5 displays the 50 emergency interventions done in 35 (24.3%) patients. Twenty-two (62.9%) had abnormal findings on the merged vital parameter immediately before the CT examination strategy decision. Seven (20%) underwent CT within 1.5 h (six SWBCT).

**Table 5:** Emergency interventions within 24 h after the accident in patients admitted with TTA

Total number of emergency interventions ^a^ per age group	< 5 years *n* = 4 (%)	5–16 years n = 3 (%)	> 16 years *n* = 43 (%)	Total number *n* = 50 (%)
Type of emergency intervention ^a^ within 24 h after the accident ^b^				
Burn wound interventions in OR	0	0	1 (2.3)	1 (2.0)
Chest tube	0	1 (33.3)	11 (25.6)	12 (24.0)
Emergency laparotomy	0	0	4 (9.3)	4 (8.0)
Intracranial pressure monitoring	0	1 (33.3)	8 (18.6)	9 (18.0)
Craniotomy/ectomy	0	0	4 (9.3)	4 (8.0)
Active external rewarming	3 (75.0)	1 (33.3)	5 (11.6)	9 (18.0)
Active internal rewarming with ECMO	1 (25.0)	0	1 (2.3)	2 (4.0)
Interventional angiography	0	0	4 (9.3)	4 (8.0)
Other emergency interventions ^c^	0	0	5 (11.6)	5 (10.0)
Total interventions	4	3	43	50

*TTA* Trauma team activation, *OR* operating room, *ECMO* extracorporeal membrane oxygenating, ^a^ as defined in the Norwegian trauma register manual added active external and internal rewarming, ^b^ one patient can have several interventions, ^c^ includes arterial and venous suture, amputation, cranium fracture debridement

Eleven (31.4%) were transported to the OR without preoperative CT. Two (18.2%) underwent laparotomies, three (27.2%) operations for arterial bleedings in extremities, two (18.2%) chest tube insertions and rewarming, three (27.3%) rewarming, and one (9.1%) burn injury treatment. Among the 52 patients with ISS > 15, 30 (57.7%) had an emergency intervention and five (16.7%) of these 30 died within 30 days after the accident. In addition, 23 (15.6%) patients were intubated pre-hospitally and another eight (5.5%) within the first 24 h.

Table 6 grades the injuries detected with CT and the use of emergency interventions in the three CT examination strategy groups. The proportion undergoing emergency intervention was significantly lower in the selective CT group (7%) compared to the SWBCT group (29%, *p = 0.005*) and the no CT group (39%, *p = 0.001*). Among the 73 patients in the SWBCT group, 17 (23.3%) had no or only AIS 1 injuries detected on CT.

**Table 6:** Distribution of CT use, emergency interventions and CT detected injuries in patients admitted with TTA

Type of CT diagnostics done during trauma admissions	No CT, *n* = 28		SWBCT, *n* = 73		Selective CT, n = 43			Total
Intervention within 24 h after the accident^a^	Emergency intervention	No emergency intervention	Emergency intervention	No Emergency intervention	Emergency intervention		No emergency intervention	
, n	11 (39%)	17 (61%)	21 (29%)	52 (71%)	3 (7%)		40 (93%)	144
Trauma admission CT finding groups								
High injury grade group, (AIS ≥ 2 in ≥2 CT body areas), n			15 (71.4%)	18 (34.6%)	1 (33.3%)		3 (7.5%)	37
Moderate injury grade group, (AIS ≥ 2 in one CT body area), n			3 (14.3%)	20 (38.5%)	2 (66.6%)		17 (42.5%)	42
Low injury grade group, (no injuries or only AIS 1 injuries in CT body areas), n			3 (14.3%)	14 (26.9%)	0 (0%)		20 (50.0%)	37
No findings on TA CT and no intervention in CT examined area, in low injury grade group, n			3 (100.0%)	10 (71.4%)	0 (0%)		13 (65.0%)	26

*CT* Computer tomography *TTA* Trauma team activation, *TA* trauma admission ^a^Emergency intervention group includes patients with emergency interventions as listed in the Norwegian trauma register manual added active external and internal rewarming, the no emergency intervention group include patients with non emergency interventions or no intervention

In addition to the emergency interventions, another 359 interventions were done. In total, 409 interventions were undertaken in 118 (81.9%) patients. Two hundred and seventy-seven (67.7%) of the interventions were in a CT examined area.

### Children versus adults

When comparing children to adults, the proportion undergoing CT examination was significantly lower both during trauma admissions (53.8% versus 86.4%, *p < 0.001*) and the total hospitalization (61.5% versus 89.8%, *p < 0.001*). The proportion of injuries detectable with CT (ISS 4–75) (65.4% versus 73.7%, *p = 0.64*) and the proportion undergoing interventions during the first 24 h (80.9% versus 75.4%, *p = 0.561*) were not significantly different between children and adults.

### Possible predictors for SWBCT

Table 7 shows associations between patient and trauma characteristics, vital parameters, blood values and CT examination strategy in 113 adults. The five hemodynamically unstable adults sent directly to the operation room without CT and the 26 children were excluded from this analysis. In unadjusted logistic regression analysis, three parameters were associated (*p* < 0.05) with use of SWBCT: GCS < 13 or intubated, transported with physician to trauma admission and high-energy trauma mechanism. These were included in the adjusted analysis. It showed that patients with a high-energy trauma mechanism had significantly (*p = 0.028*) increased odds compared to low energy trauma (odds ratio = 4.39, 95% CI 1.174–16.413) for being examined with a SWBCT. Patients with GCS < 13 or intubated also had increased odds (odds ratio = 2.448, CI 0.912–6.574) for this examination.

**Table 7:** Objective clinical parameters in 113 adult patients during trauma admissions, univariable and multivariable logistic regression^a^

Clinical data just before trauma admission CT examination strategy decision	SWBCT (*n* = 66)	Other choices ^b^ (*n* = 47)	Missing values “SWBCT” group	Missing values “other choices” group	Unadjusted OR (95% CI)	p-value*	Adjusted OR (95% CI)	p-value*
Scale variables	mean (SD)	mean (SD)	n	n				
Age	41.9 (16.9)	40.2 (18.0)	0	0	1.003 (0.981–1.025)	0.799		
Heart rate, beats/minute	86.7 (19.7)	87.1 (21.0)	0	1	0.999 (0.980–1.018)	0.916		
Systolic blood pressure, mmHg	132.2 (26.4)	134.3 (24.3)	0	1	0.997 (0.982–1.012)	0.670		
Shock index (HR/SPB)	0.7 (0.3)	0.7 (0.2)	0	1	1.785 (0.300–10.607)	0.524		
Respiratory rate, breath/minute	19.6 (6.8)	20.0 (4.5)	18	4	0.989 (0.921–1.062)	0.758		
Blood haemoglobin g/dl	14.2 (1.8)	14.5 (1.2)	1	1	0.887 (0.691–1.138)	0.346		
Blood lactate mmol/l	2.2 (1.8)	1.7 (1.4)	22	14	1.220 (0.899–1.656)	0.202		
Blood base excess mmol/l	−2.9 (3.3)	−2.3 (3.8)	19	15	0.953 (0.832–1.091)	0.484		
Minutes from accident to start of first CT	237 (207)	217 (190)	0	11	1.001 (0.998–1.003)	0.626		
Categorical variables	n (%)	n (%)	n	n				
Male sex	55 (83.3)	40 (85.1)	0	0	1.143 (0.407–3.206)	0.800		
GCS < 13 or intubated	22 (33.3)	7 (14.9)	0	0	2.857 (1.103–7.404)	0.031	2.448 (0.912–6.574)	0.076
Positive finding on chest X-ray	17 (25.8)	7 (14.9)	4	7	1.781 (0.663–4.784)	0.252		
Positive finding on pelvic X-ray	7 (10.6)	1 (2.1)	15	14	5.091 (0.596–43.452)	0.137		
Positive EFAST	7 (10.6)	4 (8,5)	10	10	1.179 (0.319–4.348)	0.805		
Transport with physician to trauma admission	46 (69.7)	25 (53.2)	0	0	2.024 (0.931–4.402)	0.075	1.767 (0.785–3.977)	0.169
High energy mechanism of trauma ^c^, n(%)	17 (25.6)	3 (6.4)	0	0	5.088 (1.396–18.543)	0.014	4.390 (1.174–16.413)	0.028

*SWBCT* Standardized whole body computer tomography*, CT* Computer tomography*, OR* Odds ratio*, CI* Confidence interval*, SD* standard deviation*,* ^a^ excluded 26 children and five adults hemodynamically unstable sent directly to the operation room from the 144 patients included in the study, ^b^ Other choices (selective CT and no CT), ^c^ following the trauma activation criteria for mechanism of trauma at the University Hospital of North Norway dichotomized as high or low energy trauma, * statistically significant (*p* < 0.05)

## Discussion

The main findings in the present study of patients admitted with TTA were that most had at least one AIS ≥ 2 injury (72.9%), underwent a CT examination (84.7%) and an intervention (81.9%). Few had AIS ≥ 2 injury in more than two ISS body areas (18.1%) and (32.4%) had abnormal vital parameters. In the selective CT group, only two (4.6%) patients underwent a complement CT, and no new injuries were identified. Children underwent significantly fewer CT examinations than adults, despite similar injury grades and use of interventions. Information about a high-energy trauma mechanism was the only parameter identified to significantly increase the odds for undergoing a SWBCT.

According to ICRP, an ionizing radiation examination is justified on level three if the potential benefit for the patient outweighs the potential risk for ionizing radiation harm. In the present study, the CT examination strategy was individualized, but the high proportion of patients with no or only minor injuries detected in the SWBCT group and the significantly lower use of CT among children, indicate that use of a selective CT examination strategy in a higher proportion of our patients would have approximated the ICRP’s level three justification principle better. The trauma team meets potentially severely injured patients in the ED. An unconscious circulatory stable patient may show no visible signs of trauma while the CT identifies several injuries. An awake and afraid patient can show symptoms indicating severe injuries while the CT shows no injuries. According to ICRP’s justification level three both are justified. In our study, 23.3% of the patients in the SWBCT group had only minor or no injuries. In previous studies, this proportion range from 14% in a study with strict criteria up to 60% in studies with liberal criteria for SWBCT [31, 62, 63]. Hence, a prospective study assessing whether SWBCT examinations are justified in individual patients, would imply registrations of the injuries suspected by the trauma team before the CT examination strategy decision. To our knowledge, such studies have not been published.

Demographics, injury pattern, use of emergency interventions and time from trauma admission to start of the first CT scan in our population mainly compares to previously published similar studies [62–65]. The median pre-hospital transportation time of nearly two hours is, however, long when compared to large urban area trauma populations, but comparable to the context in other rural populations in for example Canada [66]. In this setting, the use of immediate SWBCT as advocated by e.g. Huber-Wagner et al. [16, 18, 67, 68] cannot be justified because the long observation time provides time for clinical observation, supporting a selective CT examination strategy, at least in conscious patients [22].

Further, comparison between complete trauma centre case series, like ours, and registry-based studies including only severely injured patients (ISS > 15) may cause biased inferences. In the latter, the pre-test likelihood of an unsuspected injury is high implying that widespread use of immediate SWBCT may be justified. Interestingly, two different analyses of patients included in the TraumaRegister DGU® of the German trauma society concluded conflictingly with regard to the potential survival benefit of immediate SWBCT [16, 18, 69].

The alternative to immediate SWBCT as a screening for injuries would be a clinical decision tool providing evidence based selection criteria for CT examination strategy decision. Hare et al. [70] reviewed the literature to clarify whether such tools improve diagnostic accuracy of whole body CT, and concluded that the evidence to support this is limited. All identified studies were retrospective analyses of predictors for CT findings [32, 34, 65, 71]. Davis et al. [65] recorded all findings from clinical examination, including superficial physical signs as bruising, tenderness and swelling. They suggested a decision rule based on physical signs, vital parameters and information about the mechanism of injury. We found that information about a high-energy trauma mechanism increased the odds for being examined with a SWBCT. This is consistent with the recommendations suggested by Davis et al. [65]. To our knowledge, proposed decision rules have not been validated in prospective observational studies or evaluated against alternative strategies, such as immediate SWBCT, in randomized trials.

We found no differences in the use of duplicated CT between patients undergoing SWBCT and those examined with a selective CT strategy, and the frequency of complement examinations in the selective CT examination strategy group was low. The frequency of new findings causing an active intervention was low and not different between the groups. This indicates that the selective strategy practiced by our trauma teams is safe, despite not following a validated decision rule. This is in accordance with some previous studies [23, 63], while other report risk for missing potentially important injuries with this approach [32, 33]. The interpretation of our findings should, however, take into consideration that the use of SWBCT was relatively high (50.7%).

### Strengths and limitations

The major strength of the present study is the rigorous registration of all injuries, imaging and interventions in a clinical audit design. Further, it is a strength that the study contribute data from a rural Level 1 trauma centre, which highlights that results from large urban centres cannot be generalized without considering the geographical context.

The most important limitation is the low number of patients, implying a risk for type 2 errors. For example, the true proportion of injuries missed with the selective CT examination strategy could be higher than identified by us. In addition, variables not registered in our study could influence decisions about CT examination strategy. Further, any study of the justification of CT use requires registration of the possible injuries suspected immediately before a CT examination strategy decision is reached. The retrospective design precludes retrieval of such data.

## Conclusion

In the present study, most patients had long pre-hospital transportation times, few were admitted with abnormal vital parameters and few were injured in more than two body regions. The CT examination strategy was individualized. The high proportion of patients with no or only minor injuries detected in the SWBCT examination strategy group and the significantly lower use of CT among children, indicate that use of a selective CT examination strategy in a higher proportion of our patients would have approximated the ICRP’s justification level three, the individual dose limitation principle, better.

## Abbreviations

AIS: Abbreviated Injury Scale
CI: Confidence interval
CT: Computer tomography
ED: emergency department
GCS: Glasgow coma scale
HR: Heart rate
ICRP: International commission on radiological protection
ISS: Injury severity score
mSv: Millisivert
NISS: New ISS
OR: Operating room
PACS: picture archiving and communication system
Q1: lower quartile
Q3: upper quartile
RIS: Radiology information system
RR: respiratory rate
SBP: Systolic blood pressure
SD: Standard deviation
SI: Shock index
SWBCT: Standardized whole body computer tomography
TTA: trauma team activation
UNN: University Hospital of North Norway
